# Long-Term Effect of Endoscopic Evacuation for Large Basal Ganglia Hemorrhage With GCS Scores ≦ 8

**DOI:** 10.3389/fneur.2020.00848

**Published:** 2020-08-14

**Authors:** Haixiao Liu, Xun Wu, Zhijun Tan, Hao Guo, Hao Bai, Bodong Wang, Wenxing Cui, Longlong Zheng, Feifei Sun, Xiaoyang Zhang, Ruixi Fan, Ping Wang, Wenting Jing, Junmei Gao, Wei Guo, Yan Qu

**Affiliations:** ^1^Department of Neurosurgery, Tangdu Hospital, The Fourth Military Medical University, Xi'an, China; ^2^Department of Pathology, University of Texas Southwestern Medical Center, Dallas, TX, United States; ^3^Department of Health Statistics, The Fourth Military Medical University, Xi'an, China; ^4^Department of Neurosurgery, The 960th Hospital, Jinan, China

**Keywords:** basal ganglia hemorrhage, neuroendoscopy, minimally invasive surgical procedures, mortality, glasgow coma scale

## Abstract

**Aims:** The surgical evacuation, including stereotactic aspiration, endoscopic evacuation, and craniotomy, is the most effective way to reduce the volume of intracerebral hemorrhage. However, credible evidence for the effects of these techniques is still insufficient. The present study explored the long-term outcomes of these techniques in the treatment of basal ganglia hematoma with low Glasgow Coma Scale (GCS) scores (≤8) and large-volume (≥40 ml), which were predictors of high mortality.

**Methods:** Two hundred and fifty-eight consecutive patients were reviewed retrospectively. The primary and secondary outcomes were 6-months mortality and 6-months modified Rankin Scale score, which were assessed by a multivariate logistic regression model.

**Results:** Compared with the endoscopic evacuation group, the mortality was significantly higher in the stereotactic aspiration group (OR 6.858, 95% CI 3.146–14.953) and open craniotomy group (OR 3.315, 95% CI 1.497–7.341). Age (OR = 2.237, 95% CI 1.290–3.877) and herniation (OR = 2.257, 95% CI 1.172–4.348) were independent predictors for mortality. No significant difference in the neurological functional outcome was found in the stereotactic aspiration group (OR 0.501, 95% CI 0.192–1.308) and the craniotomy group (OR 0.774, 95% CI 0.257–2.335) compared with the endoscopic evacuation group.

**Conclusion:** Endoscopic evacuation significantly decreased the 6-months mortality in patients with hemorrhage ≥40 ml and GCS ≤ 8.

## Introduction

Spontaneous intracerebral hemorrhage (sICH) is the most common type of hemorrhagic stroke, accounting for 10–15% of all strokes (1), with an estimated mortality rate of >30% within the initial 30 days and the vast majority of survivors being disabled at 6 months (2).

Accumulated evidence has shown that large hematoma and low Glasgow Coma Scale (GCS) scores are strong predictors of high mortality and poor outcome in sICH (3, 4). Gertrude et al. found that patients with a hematoma volume ≥ 60 ml and GCS score ≤ 8 had a predicted 30-days mortality rate of 91%, whereas the predicted 30-days mortality rate of patients with a hematoma volume ≤ 30 ml and GCS score ≥ 9 was only 19% (5). Although the surgical evacuation had been used as an effective way to reduce the hematoma volume, its long-term effects are still controversial.

The Surgical Trial in Intracerebral Hemorrhage (STICH) I and STICH II studies (6, 7) indicated that there were no overall clinical benefits from early surgical evacuation compared with initial conservative management for sICH patients. However, 26% of patients initially assigned to conservative management ultimately underwent craniotomy. With reanalysis of the data from the STICH studies, patients with larger ICH volume were found to be more likely to benefit from surgery (8). Even more, the main surgical technique used in STICH I and STICH II was opening craniotomy, which might be more invasive than the minimally invasive techniques.

Recently, a slight decrease in mortality attributable to the surgical intervention was reported in the results of the Minimally Invasive Surgery Plus rt-PA for Intracerebral Hemorrhage Evacuation (MISTIE) phase III trial (9). Moreover, the residual hematoma volume affected functional outcome (10). This evidence suggests that the appropriate patients might benefit from minimally invasive evacuation. However, for now, no clinical studies compared the long-term outcomes of minimally invasive approaches with the opening craniotomy approaches directly.

In the present study, the records of sICH inpatients in Tangdu hospital admitted between January 1, 2015, and June 30, 2018 were reviewed. Finally, a retrospective analysis of the 6-months clinical outcomes was conducted in patients with hemorrhage volume ≥ 40 ml and GCS score ≤ 8. The long-term effects of the opening craniotomy and minimally invasive techniques, including stereotactic aspiration and endoscopy, were evaluated.

## Methods

### Study Design and Population

The present study was designed to compare the 6-months mortality rate and modified Rankin Scale (mRS) score of spontaneous basal ganglia hematoma patients with an initial GCS score ≤ 8 who underwent treatment with one of the following three surgical approaches: stereotactic aspiration, endoscopic evacuation, and open craniotomy. This study was approved by the Biological and Medical Ethics Committee of Tangdu Hospital (No. TDLL-2014115) and performed in Tangdu Hospital strictly following the Declaration of Helsinki (2013). All participants or relatives gave their informed consents to participate in the present study. The medical records of all patients with sICH at Tangdu Hospital between January 1, 2015, and June 30, 2018, were reviewed retrospectively.

Patients were selected according to the following criteria.

**Inclusion criteria:**

1. Diagnosis of intracerebral hemorrhage in the primary basal ganglion region by computerized tomography (CT).
2. Hematoma volume ≥ 40 ml.
3. Initial GCS score ≤ 8.
4. 18–80 years of age.
5. Treatment with stereotactic aspiration, endoscopic evacuation, or craniotomy.
6. Admission within 24 h of ictus.

**Exclusion criteria:**

1. Intracerebral hemorrhage caused by tumor, arteriovenous malformation, aneurysm, or coagulopathy.
2. Traumatic brain injury.
3. Multiple intracerebral hemorrhages.
4. Advanced dementia or disability before onset.
5. Concurrent serious illness that would interfere with the safety assessments, including hepatic, renal, gastroenterological, respiratory, cardiovascular, endocrinological, immunological, or hematological disease.
6. Malignant disease or a life expectancy of fewer than 6 months due to comorbidities.
7. Concurrent serious infectious disease (human immunodeficiency virus, tuberculosis, etc.).
8. Concurrent coagulation disorders or the use of antiplatelet or anticoagulant drugs.
9. Pregnant or lactation.
10. Participation in another simultaneous trial of intracerebral hemorrhage treatment.
11. Refusal of follow-up.

### Treatment

All patients were admitted to and managed in the neurological intensive care unit (NICU) with best medical treatment and care according to the recommendations of the American Heart Association/American Stroke Association (AHA/ASA) (11) and European Stroke Organization (ESO) (12), until they could be transferred to a general ward. CT scans, routine blood test, coagulation function tests, biochemical examinations, and other necessary examinations were performed immediately after admission. The personal and family medical histories were recorded, and a neurological physical examination was performed immediately after admission.

In the clinical treatment process, surgery approaches were selected by the surgeons according to the location, volume, and progression of the hematoma, the doctors' experience, and the patient's general condition.

### Surgical Procedures

All surgeries were conducted by a well-trained surgical team. The surgical technique was determined according to the location, volume, and progression of the hematoma, the surgeons' experience, and the patient's general condition. The principles of minimally invasiveness were followed. The surgery procedures were based on the methods described in previous studies (13–15). The decompressive craniectomy was conducted for the patients with herniation. For the patients without herniation, the decision of decompressive craniectomy was made according to the patients' condition during the operation.

For the stereotactic aspiration group, a burr hole (1–1.5 cm diameter) was drilled and a soft catheter was inserted along the long axis into the hematoma cavity under general anesthesia. Clot aspiration was done with a 5 mL handheld syringe until the first resistance. Postoperative CT was done to confirm the positioning of the catheter and stability of the residual hematoma. Then, Urokinase was injected directly into the clot through the catheter, at 10,000–30,000 units in 2 mL followed by 3 mL flush every 12 h, for up to 3 days.

For the endoscopic evacuation group, skin incision and a 3–5 cm diameter bone flap were made under general anesthesia according to the position of the hematoma showed on CT scan. The dura mater was coagulated and incised in a cruciate fashion. After the puncturing cannula was positioned in the predetermined center of the hematoma, the cannula core was removed. Clot aspiration was done with a 5 mL handheld syringe to reduce intracranial pressure. Then, the transparent sheath was introduced along the puncturing cannula to the bottom of the hematoma. Irrigation and aspiration were repeated to evacuate the hematoma through the transparent sheath.

For the craniotomy group, a traditional craniotomy was performed. After opening the dura, the neurosurgeon accessed the hematoma cavity via a transcortical/transtemporal approach under microscopic assistance and evacuate the hematoma. Decompressive craniotomy and tracheotomy were conducted if the neurosurgeon considered necessary. All the patients received the best medical treatment after surgery. A postoperative CT scan was performed 24 h after surgery to evaluate the residual hematoma for every patient.

### Data Collection and Outcomes Evaluation

Basic information (sex, age, diagnosis, etc.) was obtained from the patient information management department of our hospital. Disease history (smoking, diabetes, hypertension, and craniocerebral disease) and treatment information (surgical technique, decompressive craniectomy, and tracheotomy) were collected from the inpatient medical record system of our hospital. The preoperative status of the patients was assessed by the preoperative GCS score, herniation, the time interval between onset and surgery and the hematoma volume, which were also obtained from the inpatient medical record system.

The hematoma volume was calculated from CT scans using the formula A^*^B^*^C/2, where A is the greatest diameter on the largest hemorrhage slice, B is the maximal diameter perpendicular to A, and C is the vertical hematoma depth.

The participants were followed by calling to record their prognostic information at 1, 3, and 6 months after surgery. The primary outcome was the 6-months mortality rate after surgery. The secondary outcome was the 6-months neurological functional status, which was evaluated by mRS score. The researchers, who collected the information, and the statistician, who conducted the statistical analysis were blinded to the treatments the subjects received.

### Statistical Analysis

Given the selection bias inherent to retrospective observational studies, the Chi-squared test was used to test the intergroup balance of the possible confounding factors (Table 1). A *P*-value of <0.1 was considered unbalanced. Univariate analysis was used to determine which unbalanced variables should be included in the multivariate logistic regression model (Table 2). The multivariate logistic regression model was used to analyze the independent effect of the surgical technique on the outcome (Table 3).

**Table 1:** The inter-group balance tests of possible confounding factors among different surgical techniques.

		**Total sample (*****n*** **=** **258)**					**Survived sample (*****n*** **=** **127)**				
		**Total**	**SA**	**EE**	**OC**	***P*-value**	**Total**	**SA**	**EE**	**OC**	***P*-value**
			**(*n* = 99)**	**(*n* = 60)**	**(*n* = 99)**			**(*n* = 34)**	**(*n* = 48)**	**(*n* = 45)**	
Gender	Male	157	57 (36.3%)	39 (24.8%)	61 (38.9%)	0.6365	75	18 (24.0%)	29 (38.7%)	28 (37.3%)	0.6875
	Female	101	42 (41.6%)	21 (20.8%)	38 (37.6%)		52	16 (30.8%)	19 (36.5%)	17 (32.7%)	
Age (years)	<60	140	44 (31.4%)	39 (27.9%)	57 (40.7%)	0.0292^*^	82	21 (25.6%)	34 (41.5%)	27 (32.9%)	0.5089
	≥60	118	55 (46.6%)	21 (17.8%)	42 (35.6%)		45	13 (28.9%)	14 (31.1%)	18 (40.0%)	
Smoking	Yes	74	21 (28.4%)	22 (29.7%)	31 (41.9%)	0.0860^*^	40	7 (17.5%)	17 (42.5%)	16 (40.0%)	0.2780
	No	184	78 (42.4%)	38 (20.7%)	68 (37.0%)		87	27 (31.0%)	31 (35.6%)	29 (33.3%)	
Diabetes	Yes	19	9 (47.4%)	5 (26.3%)	5 (26.3%)	0.5241	7	2 (28.6%)	1 (14.3%)	4 (57.1%)	0.3539
	No	239	90 (37.7%)	55 (23.0%)	94 (39.3%)		120	32 (26.7%)	47 (39.2%)	41 (34.2%)	
Hypertension	Yes	215	85 (39.5%)	51 (23.7%)	79 (36.7%)	0.4806	107	29 (27.1%)	40 (37.4%)	38 (35.5%)	0.9706
	No	43	14 (32.6%)	9 (20.9%)	20 (46.5%)		20	5 (25.0%)	8 (40.0%)	7 (35.0%)	
History of craniocerebral disease	Yes	28	13 (46.4%)	5 (17.9%)	10 (35.7%)	0.6118	9	4 (44.4%)	3 (33.3%)	2 (22.2%)	0.4364
	No	230	86 (37.4%)	55 (23.9%)	89 (38.7%)		118	30 (25.4%)	45 (38.1%)	43 (36.4%)	
Herniation	Yes	84	18 (21.4%)	10 (11.9%)	56 (66.7%)	<0.0001^*^	31	2 (6.5%)	6 (19.4%)	23 (74.2%)	<0.0001^*^
	No	174	81 (46.6%)	50 (28.7%)	43 (24.7%)		96	32 (33.3%)	42 (43.8%)	22 (22.9%)	
Interval between onset and operation (hours)	≤8	88	34 (38.6%)	12 (13.6%)	42 (47.7%)	0.0153^*^	34	9 (26.5%)	9 (26.5%)	16 (47.1%)	0.1875
	>8	170	65 (38.2%)	48 (28.2%)	57 (33.5%)		93	25 (26.9%)	39 (41.9%)	29 (31.2%)	
Hematoma volume (ml)	40–80	151	68 (45.0%)	40 (26.5%)	43 (28.5%)	0.0005^*^	80	28 (35.0%)	31 (38.8%)	21 (26.3%)	0.0048^*^
	≥80	107	31 (29.0%)	20 (18.7%)	56 (52.3%)		47	6 (12.8%)	17 (36.2%)	24 (51.1%)	
Rehabilitation treatment	PRT	76	22 (28.9%)	24 (31.6%)	30 (39.5%)	0.3953	70	19 (27.1%)	24 (34.3%)	27 (38.6%)	0.7390
	NPRE	46	14 (30.4%)	19 (41.3%)	13 (28.3%)		43	12 (27.9%)	19 (44.2%)	12 (27.9%)	
	NRT	26	4 (15.4%)	10 (38.5%)	12 (46.2%)		14	3 (21.4%)	5 (35.7%)	6 (42.9%)	
Decompressive craniectomy	Yes	100	6 (6.0%)	11 (11.0%)	83 (83.0%)	<0.0001^*^	48	2 (4.2%)	9 (18.8%)	37 (77.1%)	<0.0001^*^
	No	158	93 (58.9%)	49 (31.0%)	16 (10.1%)		79	32 (40.5%)	39 (49.4%)	8 (10.1%)	
Tracheotomy	Yes	72	21 (29.2%)	20 (27.8%)	31 (43.1%)	0.1609	47	10 (21.3%)	17 (36.2%)	20 (42.6%)	0.3751
	No	186	78 (41.9%)	40 (21.5%)	68 (36.6%)		80	24 (30.0%)	31 (38.8%)	25 (31.3%)	

*SA, Stereotactic Aspiration; EE, Endoscopic Evacuation; OC, Open Craniotomy; PRT, Professional Rehabilitation Treatment; NPRT, Un-professional Rehabilitation Treatment; NRT, No Rehabilitation Treatment*.

* *The difference has statistical significance. The bold values means that the P-values has statistical significance*.

**Table 2:** The result of the univariate analyses to explore the independent risk factors associated with mortality and low mRS score.

		**Mortality (*****n*** **=** **258)**				**Modified Rankin scale score (*****n*** **=** **127)**			
		**Total**	**Die**	**Survive**	***P*-value**	**Total**	**0–2**	**3–5**	***P*-value**
Surgical methods	SA	99	65 (65.7%)	34 (34.3%)	<0.0001^*^	34	24 (70.6%)	10 (29.4%)	0.0390^*^
	EE	60	12 (20.0%)	48 (80.0%)		48	24 (50.0%)	24 (50.0%)	
	OC	99	54 (54.5%)	45 (45.5%)		45	19 (42.2%)	26 (57.8%)	
Age (years)	<60	140	58 (41.4%)	82 (58.6%)	0.0011^*^	–	–	–	
	≥60	118	73 (61.9%)	45 (38.1%)		–	–	–	
Smoking	Yes	74	34 (45.9%)	40 (54.1%)	0.3251	–	–	–	
	No	184	97 (52.7%)	87 (47.3%)		–	–	–	
Herniation	Yes	84	53 (63.1%)	31 (36.9%)	0.0060^*^	31	11 (35.5%)	20 (64.5%)	0.0267^*^
	No	174	78 (44.8%)	96 (55.2%)		96	56 (58.3%)	40 (41.7%)	
Interval between onset and operation (hours)	≤8	88	54 (61.4%)	34 (38.6%)	0.0144^*^	–	–	–	
	>8	170	77 (45.3%)	93 (54.7%)		–	–	–	
Hematoma Volume (ml)	40–80	151	71 (47.0%)	80 (53.0%)	0.1518	80	49 (61.3%)	31 (38.8%)	0.0124^*^
	≥80	107	60 (56.1%)	47 (43.9%)		47	18 (38.3%)	29 (61.7%)	
Decompressive craniectomy	Yes	100	52 (52.0%)	48 (48.0%)	0.7542	48	20 (41.7%)	28 (58.3%)	0.0510^*^
	No	158	79 (50.0%)	79 (50.0%)		79	47 (59.5%)	32 (40.5%)	

*SA, Stereotactic Aspiration; EE, Endoscopic Evacuation; OC, Open Craniotomy*.

* *The difference has statistical significance. The bold values means that the P-values has statistical significance*.

*–Not Applicable*.

**Table 3:** The result of the multivariate logistic regression model to explore the independent risk factors associated with mortality or low mRS score.

		**Mortality**		**Modified Rankin scale**	
		**(*****n*** **=** **258)**		**score (*****n*** **=** **127)**	
		**OR**	**95% CI**	**OR**	**95% CI**
Surgical methods (reference is EE)	SA	6.858	(3.146, 14.953)	0.501	(0.192, 1.308)
	OC	3.315	(1.497, 7.341)	0.774	(0.257, 2.335)
Age (year) (reference is <60)	≥60	2.237	(1.290, 3.877)	–	–
Herniation (reference is no)	Yes	2.257	(1.172, 4.348)	1.724	(0.648, 4.608)
Interval between onset and operation (hours) (reference is >8)	≤8	1.421	(0.785, 2.572)	–	–
Hematoma volume (ml) (reference is 40–80)	≥80	–	–	2.044	(0.930, 4.495)
Decompressive craniectomy (reference is no)	Yes	–	–	0.682	(0.243, 1.912)

*SA, Stereotactic Aspiration; EE, Endoscopic Evacuation; OC, Open Craniotomy*.

*–Not Applicable*.

The mRS score was dichotomized as a poor outcome (mRS score = 3–5) and a favorable outcome (mRS score = 0–2). The Chi-squared test was used to analyze the categorical variables. The hospital stay was expressed as Mean ± SEM. The unpaired *t*-test and one-way ANOVA test were used for intergroup comparison. Statistical significance was assumed with *P* < 0.05. All analyses were conducted using SAS/STAT version 9.4 (SAS Company, Cary, NC).

## Results

### Patient Numbers

**Figure 1:**
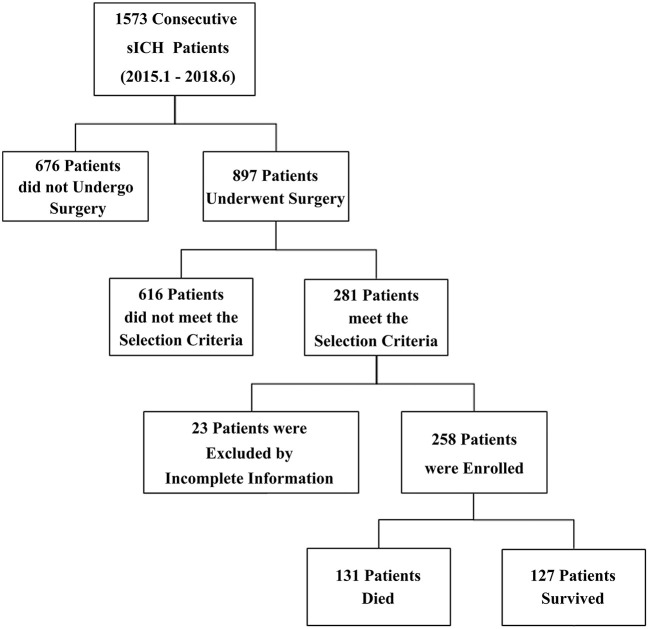
Enrollment in this study. A total of 1,573 consecutive sICH patients were reviewed. A total of 258 patients, including 131 non-survivors and 127 survivors, were enrolled according to the selection criteria.

A total of 1,573 consecutive sICH patients admitted between January 1, 2015, and June 30, 2018 were reviewed retrospectively. Finally, 258 patients with volume ≥ 40 ml and GCS score ≤ 8 were enrolled according to the inclusion and exclusion criteria. The patient numbers at each stage of study were shown in a flow diagram (Figure 1).

### Basic Characteristics

Information about gender, age, smoking, diabetes, hypertension, history of craniocerebral disease, herniation, interval between onset and operation, hematoma volume, rehabilitative treatment, decompressive craniectomy, and tracheotomy was collected (Table 1).

Intergroup equilibrium analysis was conducted to identify the potential confounding factors. Age (*P* = 0.0292), smoking (*P* = 0.0860), herniation (*P* < 0.0001), time interval between onset and surgery (*P* = 0.0153), hematoma volume (*P* = 0.0005), and decompressive craniectomy (*P* < 0.0001) were unbalanced variables in the entire cohort. However, in the surviving patients, the unbalanced variables were herniation (*P* < 0.0001), hematoma volume (*P* = 0.0048) and decompressive craniectomy (*P* < 0.0001) (Table 1).

In the stereotactic aspiration group, six patients received decompressive craniectomy. All of them were performed after the initial surgery when deterioration happened. In the endoscopic evacuation group, a total of 11 patients received decompressive craniectomy. 72.7% (8/11) of them were performed in the initial surgery, and 27.3% (3/11) of them were performed after the initial surgery when deterioration happened. In the open craniotomy group, a total of 83 patients received decompressive craniectomy. 92.8% (77/83) of them were performed in the initial surgery, and 7.2% (6/83) of them were performed after the initial surgery when deterioration happened.

### Outcome Assessment

**Figure 2:**
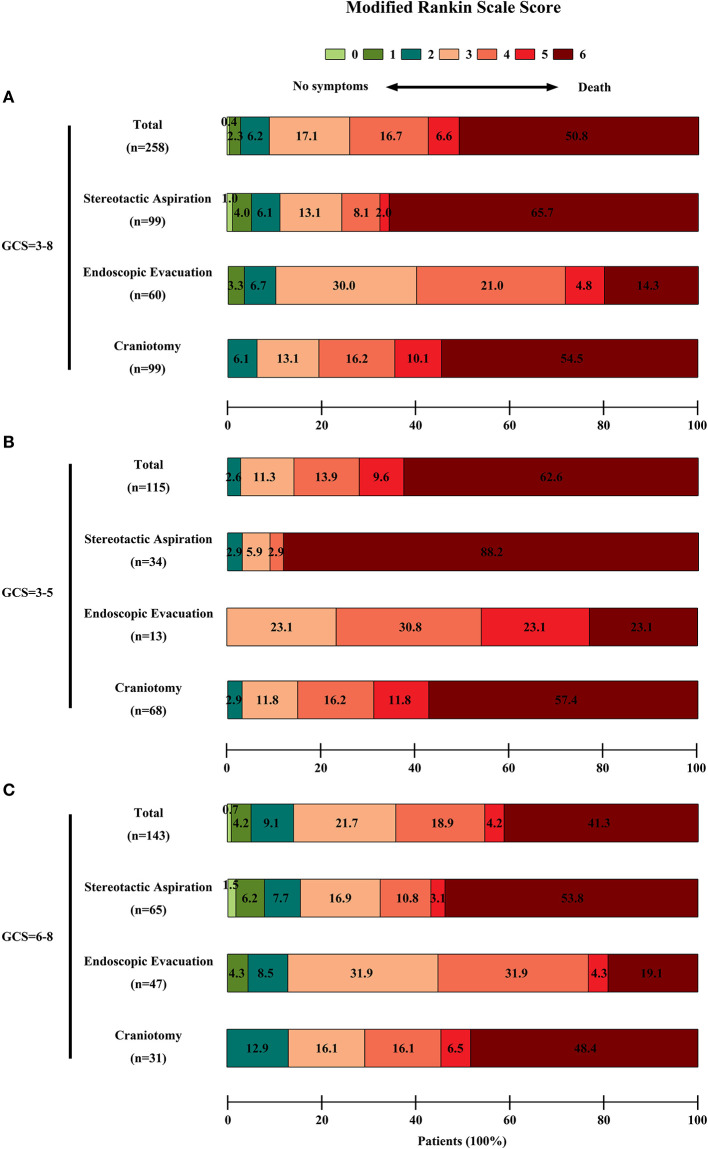
Functional outcome of patients in each group. The functional outcome was assessed by the mRS score. The scores ranged from 0 to 6, with 0 indicating no symptoms; 1, no clinically significant disability; 2, slight disability; 3, moderate disability; 4, moderately severe disability; 5, severe disability; and 6, death. A score of 3–5 was recognized as a poor outcome. The percentages of patients by score are shown in each cell. **(A)** The mRS scores of the entire cohort of patients. There were significant differences among the three groups on univariate analysis in the 6-month clinical outcomes (*P* < 0.0001 for the mortality, *P* = 0.0390 for low mRS score in surviving patients). **(B)** The mRS scores of patients with an initial GCS score = 3–5. **(C)** The mRS scores of patients with an initial GCS score = 6–8.

In the entire cohort, the overall 6-months mortality rate was 50.8% (131/258 patients). In the surviving patients, the rate of poor 6-months neurological functional outcome was 81.9% (104/127 patients). The 6-months mortality rate in the stereotactic aspiration, endoscopic evacuation, and craniotomy groups was 65.7% (65/99 patients), 20.0% (12/60 patients), and 54.5% (54/99 patients) respectively. The 6-months poor neurological functional outcome rate in survivors in the stereotactic aspiration, endoscopic evacuation, and craniotomy groups was 67.6% (23/34 patients), 87.5% (42/48 patients), and 86.7% (39/45 patients), respectively. The 6-months mortality rate and 6-months mRS scores of patients with an initial GCS score of 3–5 or 6–8 are also calculated. The mRS score distribution is shown in Figure 2.

The unbalanced characteristics were included in the univariate analysis to investigate whether they were relevant to the mortality rate or mRS score (Table 2). The potential confounding factors were age (*P* = 0.0011), herniation (*P* = 0.0060), and the interval between onset and operation (*P* = 0.0144) in the entire cohort. However, among the survivors, these factors were herniation (*P* = 0.0267), hematoma volume (*P* = 0.0124), and decompressive craniectomy (*P* = 0.0510). To minimize the influence of these confounding factors, multivariate logistic regression analysis was conducted.

Multivariate analysis showed that the mortality rate was significantly higher in the stereotactic aspiration group (OR 6.858, 95% CI 3.146–14.953) and the open craniotomy group (OR 3.315, 95% CI 1.497–7.341) than in the endoscopic evacuation group. Age (OR 2.237, 95% CI 1.290–3.877) and herniation (OR 2.257, 95% CI 1.172–4.348) were independent predictors for mortality (Table 3). Interval Between Onset and Operation (OR 1.421, 95% CI 0.785–2.572) showed no independent association with functional recovery (Table 3).

The neurological functional status in the stereotactic aspiration group (OR 0.501, 95% CI 0.192–1.308) and the craniotomy group (OR 0.774, 95% CI 0.257–2.335) was not significantly different from that in the endoscopic evacuation group. However, the patients with a hematoma volume of ≥ 80 ml had a trend of higher mRS scores (HR 2.044, 95% CI 0.930–4.495) than the patients with a hematoma volume = 40–80 ml. Herniation (HR 1.724, 95% CI 0.648–4.608), and Decompressive Craniectomy (HR 0.682, 95% CI 0.243–1.912) showed no independent association with functional recovery (Table 3).

In the survivors, the average length of a hospital stay is 11.18 ± 1.167, 12.52 ± 0.80, and 15.45 ± 0.81 days in the stereotactic aspiration, endoscopic evacuation, and craniotomy group, respectively. The stereotactic aspiration group (*P* = 0.0027) and endoscopic evacuation group (*P* = 0.0117) have a shorter length of hospital stay than the craniotomy group.

The difference in reoperation rate among these three groups had no statistical significance. In the entire cohort, 18 (7.0%) patients required reoperation, which included 7 (7.1%) in the stereotactic aspiration group, 4 (6.7%) in the endoscopic evacuation group, and 7 (7.1%) in the craniotomy group.

## Discussion

Stroke is the second most common cause of death worldwide (16). Epidemiological studies have revealed that China has the highest incidence of hemorrhagic stroke worldwide (17, 18). The typical regions of sICH are the deep gray matter structures, including the basal ganglia and thalamus (1). sICH in these regions causes death or dependency in more than 70% of patients (19).

The treatment of patients with sICH remains controversial. Conservative management and surgical evacuation are the main treatment choices for sICH. The surgical evacuation is an effective way to reduce the hematoma volume, and the stereotactic aspiration, endoscopic evacuation, and craniotomy are the most techniques used in such conditions. Multiple clinical trials comparing surgical to conservative management have failed to demonstrate a clear clinical benefit of hemorrhage evacuation (20).

Recently, the results of the MISTIE III trial were published. The primary results of MISTIE III showed that the proportion of patients with a good functional outcome was not significantly higher in the surgical treatment group (44.2%) than in the medical treatment group (41.7%) (9). Interestingly, the secondary endpoints indicated a slight decrease in mortality attributable to surgical intervention. Moreover, the residual hematoma volume affected the proportion of surgically-treated patients with a good functional outcome. Among 145 surgically-treated patients with a residual hematoma volume of ≤ 15 mL at the end of treatment, 53.1% achieved a good functional outcome, compared with 32.7% of the 101 surgically-treated patients with a residual volume of > 15 mL (10).

It is believed that different treatment strategies might be suitable for different groups of patients with particular characteristics (such as GCS score, hematoma volume, and lesion location). However, subgroup analysis is inadequate till now, which might lead to an underestimate of the impact of confounding factors (21). For the patients with low GCS score and large hematoma, surgical evacuation, including minimally invasive surgery and craniotomy, are recommended to improve survival (22). However, there is a lack of clinical evidence about how early-stage surgery can be beneficial for the patients with initial GCS score ≤ 8 till now. Only 24.65 and 20% of patients have an initiate GCS score ≤ 8 in the STICH study and MISTIE study. Whereas, 59% of patients have an initiate GCS score ≤ 8 in our center (23).

Recently, with the development of less invasive surgical techniques, minimally invasive approaches, including the endoscopic evacuation and stereotactic aspiration, had shown several advantages over the traditional open craniotomy (24). However, credible evidence for the effect of these techniques was still insufficient. A recent study showed that patients with hematoma volume > 40 mL were more likely to have a favorable outcome from surgical evacuation than conservative treatment (25). Besides, our previous study compared the long-term outcomes of the three surgical techniques in the treatment of spontaneous basal ganglia hemorrhage. According to the subgroup analysis stratified by hematoma volume, endoscopic aspiration can decrease the 6-months mortality of spontaneous basal ganglia hemorrhage, especially in patients with a hematoma volume ≥ 40 ml (23). Thus, the present study explored the long-term outcomes of endoscopic evacuation, stereotactic aspiration, and craniotomy in the treatment of large basal ganglia hematoma patients (≥40 ml) with initial GCS ≤ 8.

In our series, the overall 6-months mortality rate for the patients with a hematoma volume of ≥40 ml and GCS score ≤ 8 was 50.78%. More importantly, the 6-months mortality rate in the endoscopic evacuation group was 20.00%, which was significantly lower than that in the stereotactic aspiration and craniotomy groups.

The result of the present study showed that herniation occurred in 32.6% (84/258) patients with the hematoma volume ≥40 ml and GCS score ≤ 8. Meanwhile, herniation has been demonstrated to be one of the most critical independent indicators of high mortality in the present study. Thus, early surgical intervention is recommended for these patients to avoid herniation and reduce mortality.

Sufficient evidence has demonstrated that decompressive craniectomy plus external ventricular drainage is suitable for patients with large hematomas and low GCS scores with a significant midline shift (3, 11). Interestingly, in the present study, 83/99 (83.84%) patients underwent decompressive craniectomy in the open craniotomy group, whereas only 11/60 (18.33%) patients underwent decompressive craniectomy in the endoscopy group. These results suggest that endoscopic evacuation could be the optimal surgical choice and an effective lifesaving method for patients with hematoma volume ≥ 40 ml and GCS score ≤ 8.

Brain injury caused by ICH can be described as a biphasic process. Primary brain injury causes direct traumatic damage to regional neurons (26). More importantly, intracerebral hematoma initiates a cascade of secondary injuries leading to irreversible neuronal damage, inadequate cerebral blood flow, increased intracranial pressure, and herniation (27). Clinical evidence has shown that secondary brain injury causes a higher risk of death than primary injury (28).

Hematoma removal surgery is speculated to be an appropriate ICH treatment option because it can alleviate the mass-effect of the hematoma and reduce neurotoxic blood degradation products. Thus, these surgeries can lower intracranial pressure, improve perfusion, alleviates brain edema, and attenuate neuronal damage. However, the surgical procedure brings new damage to the patient meanwhile. The net benefits brought by surgery removal for spontaneous ICH patients remains controversial. In the present study, the net benefit of endoscopic surgery is more than the open craniotomy and stereotactic aspiration in the basal ganglia hemorrhage patients with GCS score ≤ 8 and hemorrhage volume ≥ 40 ml. The present results indicate that endoscopic evacuation could effectively alleviate secondary brain injury, resulting in a decreased mortality rate. However, as indicated by our data, no significant difference in the neurological outcome of the survivors was detected among these three groups. This result suggests that primary brain injury, which is caused by direct damage to regional neurons, may not be mitigated by current surgical approaches.

Currently, the hematoma removal surgeries include open craniotomy surgery and minimally invasive surgery. Open craniotomy is the most studied approach in this clinical scenario. However, due to the deep location of basal ganglia, open craniotomy might cause iatrogenic damage to the healthy cerebral tissue (29). Comparing with open craniotomy, the minimally invasive surgery has much smaller interference to the surrounding normal brain tissues. Despite the recent progresses on the therapeutic effects, stereotactic aspiration still has its limitation. Since the drainage usually takes several days, the mass effect is relieved slowly. On the other hand, the hematoma removal efficiency is unsatisfactory in some cases, and the residual hematoma metabolites continue to cause secondary damage (30, 31).

Comparing with stereotactic aspiration, the endoscopic evacuation surgery provides the surgeon with a good visible operation field and more operating space, which makes it possible to deal with the hematoma and surrounding tissues delicately. The hematoma can be removed rapidly and thoroughly with less surgical trauma by endoscopic evacuation. Besides, the endoscopic evacuation surgery can remove the blood accumulation in the third ventricle, fourth ventricle, and lateral ventricles in some situations. Therefore, the secondary injury caused by mass effect and neurotoxic blood degradation products might be alleviated more effectively by endoscopic evacuation (32, 33). Future studies are needed to explore the relationship between the outcomes and the residual hematoma volume and perihematomal edema of different surgical techniques.

Of course, there are several limitations to the present study. First, because of its retrospective nature, the possibility of selection bias cannot be excluded. Second, potential methodological limitations might influence the results of the present study. Third, the present results are derived from a single center and have limited generalizability. Further prospective randomized trials are needed to confirm the findings of this study.

## Conclusion

Endoscopic evacuation significantly decreases the 6-months mortality rate of patients with GCS score ≤ 8 and basal ganglia ICH volume ≥ 40 ml. Besides, the low mortality rate is more likely to be achieved in patients who were under 60 years of age and without herniation. Thus, early surgical intervention is recommended for these patients to avoid herniation. These results would be helpful for neurosurgeons to decrease the mortality of sICH. However, the 6-months neurological outcome showed no difference between different surgical methods. The optimal surgical technique, intervention timing, and patient selection might allow appropriate patients to benefit from hematoma evacuation. These preliminary results warranted a further large, prospective, randomized study.

## Data Availability Statement

The original contributions presented in the study are included in the article/supplementary material, further inquiries can be directed to the corresponding author/s.

## Ethics Statement

The studies involving human participants were reviewed and approved by Biological and Medical Ethics Committee of Tangdu Hospital. Written informed consent for participation was not required for this study in accordance with the national legislation and the institutional requirements.

## Author Contributions

YQ and WG conceived and supported this research. HL and XW designed, conducted the present study, and drafted the manuscript. YQ, HG, and HB revised the manuscript. ZT and BW conducted the statistic analyze and interpreted the data. HL, WC, LZ, FS, and XZ collected the primary data. RF, PW, WJ, and JG completed the follow-up and collected the associated data. All authors of this work met ICMJE criteria for authorship, made substantial contributions to the conception and design, acquisition of data, analysis and interpretation of data, drafting, critical revising, and final approval of this manuscript.

## Conflict of Interest

The authors declare that the research was conducted in the absence of any commercial or financial relationships that could be construed as a potential conflict of interest.
